# Striking a balance: PIP_2_ and PIP_3_ signaling in neuronal health and disease

**DOI:** 10.37349/ent.2021.00008

**Published:** 2021-10-29

**Authors:** Kamran Tariq, Bryan W. Luikart

**Affiliations:** Department of Molecular and Systems Biology, Geisel School of Medicine at Dartmouth, Hanover, NH 03755, USA

**Keywords:** Phosphoinositides, cholesterol, autism, Alzheimer’s, ion channels, cytoskeleton, AKT, mammalian target of rapamycin

## Abstract

Phosphoinositides are membrane phospholipids involved in a variety of cellular processes like growth, development, metabolism, and transport. This review focuses on the maintenance of cellular homeostasis of phosphatidylinositol 4,5-bisphosphate (PIP_2_), and phosphatidylinositol 3,4,5-trisphosphate (PIP_3_). The critical balance of these PIPs is crucial for regulation of neuronal form and function. The activity of PIP_2_ and PIP_3_ can be regulated through kinases, phosphatases, phospholipases and cholesterol microdomains. PIP_2_ and PIP_3_ carry out their functions either indirectly through their effectors activating integral signaling pathways, or through direct regulation of membrane channels, transporters, and cytoskeletal proteins. Any perturbations to the balance between PIP_2_ and PIP_3_ signaling result in neurodevelopmental and neurodegenerative disorders. This review will discuss the upstream modulators and downstream effectors of the PIP_2_ and PIP_3_ signaling, in the context of neuronal health and disease.

## Introduction

Phosphoinositides are membrane phospholipids involved in regulation of key cellular processes that range from cell growth, protein metabolism, membrane remodeling, and trafficking, to cell death [1–5]. Traditionally, phosphoinositides have been characterized as precursors to secondary messengers for cellular pathways [6, 7], however, evidence for direct roles for these phospholipids in membrane dynamics is also piling up [8]. Chemically, the seven distinct species of phosphoinositides are derived from unique phosphorylations of myo-inositol head group of the phosphatidylinositol (PI) at position 3,4 and 5 hydroxyl residues, either singly or in combination [9]. This addition and removal of phosphate groups is selectively regulated by specific phosphoinositide kinases and phosphatases, which are evolutionarily well-conserved [2].

**Figure 1.**
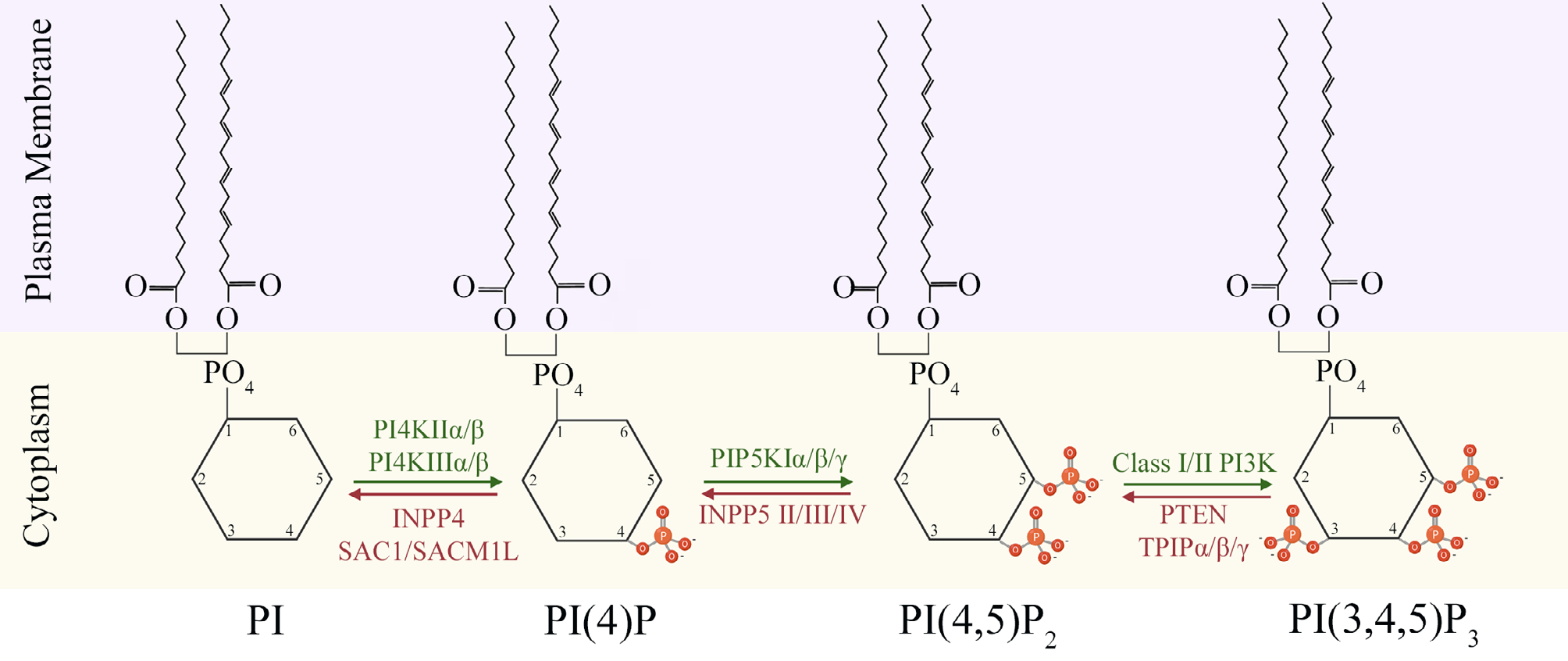
Biosynthesis of PIP_2_ and PIP_3_ at plasma membrane-cytoplasm interface. The equilibrium between synthesis of PIP_2_ and PIP_3_ at membrane-cytoplasm interface is maintained through addition of phosphate groups (orange) by kinases (green), and its removal by phosphatases (red) at position 3,4, or 5 of the cytoplasmic inositol head group of phosphoinositides (PI). INPP4: inositol polyphosphate 4-phosphatase; SACM1L: SAC1 like phosphatidylinositide phosphatase; INPP5: inositol polyphosphate 5-phosphatase; TPIP: transmembrane phosphatase with tensin homology (TPTE) and PTEN homologous inositol lipid phosphatase

PI 4,5-bisphosphate [PI(4,5)P_2_, hereon referred to as PIP_2_], and PI 3,4,5-trisphosphate [PI(3,4,5)P_3_, hereon referred to as PIP_3_] are the focus of this review. PIP_2_ and PIP_3_ are constituents of plasma membrane and their precisely regulated abundance changes in response to cell intrinsic and/or extrinsic stimuli. The PIP_2_ is primarily formed as a result of sequential phosphorylations at position 4 and position 5 hydroxyl residues of PI by PI 4-kinase (PI4K), and PI phosphate kinase (PIPK), respectively. PIP_3_ is generated from PIP_2_ by addition of a phosphate on position 3 hydroxyl residue by Class I phosphoinositide 3-kinase (PI3K). Conversely, dephosphorylation of PIP_3_ into PIP_2_ by phosphatase and tensin homolog deleted on chromosome 10 (PTEN) phosphatase also contributes towards maintenance of a PIP_2_/PIP_3_ homeostasis in the cells (Figure 1). In the nervous system, both PIP_2_ and PIP_3_ play essential roles in regulating neuronal morphogenesis, electrical activity, ion channels and neurotransmitters mediated signal transduction, synaptic plasticity, and cytoskeletal remodeling [10–13]. Perturbations to the delicate balance between PIP_2_ and PIP_3_ levels result in aberrant neurodevelopment and neurodegenerative diseases, like autism spectrum disorders (ASD) and Alzheimer’s disease (AD), among others [14, 15]. This review discusses the upstream modulators that maintain this PIP_2_/PIP_3_ balance, and downstream mediators that are influenced by it, in the context of neuronal regulation and disease.

## Modulators of PIP_2_/PIP_3_ balance

PIP_2_ and PIP_3_ are primarily distributed at the cytoplasmic leaflet of plasma membrane and their localized abundance plays diverse roles in regulation of varied cellular processes. A cell needs to maintain a critical balance of these PIs for its normal function. PIP_2_ and PIP_3_ are essential for ligand-associated receptor tyrosine kinase (RTK) and G-protein coupled receptor (GPCR) mediated signaling [16], and their cellular levels and activity can be modulated by PI kinases and PI phosphatases [17], which are described below:

### Kinases

Phosphoinositide kinases are diverse group of enzymes that perform the addition of phosphate group on the hydroxyl residues of myo-inositol ring of PIs. These kinases have substrate specificity for PIs, as well as specificity for their target hydroxyl residue on these PIs.

### PI4Ks

**Figure 2.**
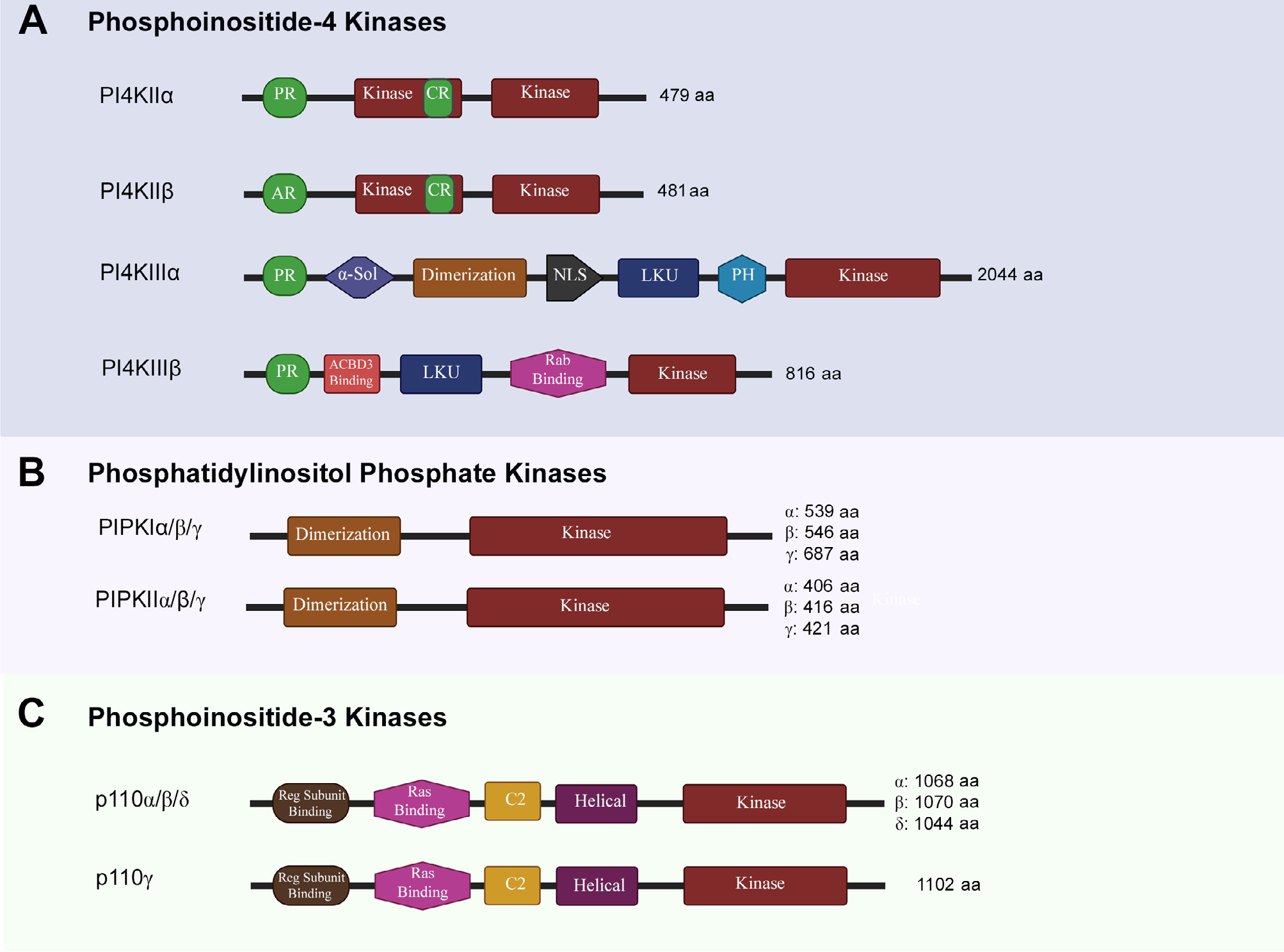
Structural features of phosphoinositide kinases. aa: amino acid; α-Sol: α-solenoid domain; NLS: nuclear localization signal; LKU: lipid kinase unique; PH: pleckstrin homology domain; ACBD3: acyl-CoA-binding protein 3; Reg: regulatory. The schematics for structural domains are not to scale

The formation of PIP_2_ is catalyzed by two sequential phosphorylations, first of which is catalyzed by PI4Ks. The two classes of PI4Ks (type II and type III) are categorized into two distinct groups of isozymes, each based on their structure, which lends itself to their specific spatiotemporal activities (Figure 2A). Type II PI4Ks are categorized into PI4KIIα (gene names are italicized and put in brackets; *PI4K2A*) and PI4KIIβ (*PI4K2B*) isozymes, while Type III PI4Ks are categorized into PI4KIIIα (*PI4KA*) and PI4KIIIβ (*PI4KB*) isozymes [18]. Structurally, type II isozymes have a bisected kinase domain, with its cystine-rich (CR) N-terminal half containing a palmitoylation site, which is likely involved in membrane tethering [19, 20]. The type II isozymes are homologous but have differences in their N-terminals. PI4KIIα contains a proline-rich (PR) region at its N-terminal, while PI4KIIβ contains an acidic region (AR) [21] (Figure 2A). PI4KIIα and PI4KIIβ are ubiquitously expressed but PI4KIIα has higher expression in brain tissue when compared to PI4KIIβ and its dysfunction is associated with late onset neurodegenerative disease in mouse models [21, 22]. Both PI4KIIα and PI4KIIβ localize to intracellular membranes and have been reported to be involved in cellular vesicle trafficking [23, 24]. The association of PI4KIIα with synaptic vesicles points to its role in neurotransmission [25].

The Type III isozyme, PI4KIIIα is primarily localized at plasma membrane and is the main source of generation of precursor PIs. Generation of PIP_2_ from these precursor PIs regulates Ca^2+^ signaling [26]. Structurally, both type III isozymes contain a N-terminal pleckstrin homology (PH) domain, a LKU domain and a continuous kinase domain on the C-terminal. PI4KIIIα contains a large N-terminal solenoid domain (α-Sol), whose role remains unknown [27]. PI4KIIIα also contains a NLS and a PH domain, implicated in plasma membrane association [28]. PI4KIIIβ contains a Rab-binding region implicated in lipid transport and membrane trafficking [29].

The activity of type III PI4Ks is specified by their association with a myriad of regulatory binding partners. PI4KIIIα directly interacts with tetratricopeptide repeat domain 7 (TTC7)A/B, with FAM126 acting as a scaffold, to form a dimer of heterotrimers [30]. TTC7 interacts with plasma membrane associated EFR3 homolog A (EFR3A) protein to activate PI4KIIIα. On the other hand, PI4KIIIβ interacts with a variety of binding partners of its own, namely Rab11 GTPase, ACBD3, and 14–3-3 regulatory proteins. The Rab11 binding localizes Rab11 to trans-Golgi network (TGN), while the interaction with ACBD3 is reported to be necessary for viral pathogenesis [31]. Protein kinase D mediated S294 phosphorylation stabilizes PI4KIIIβ by allowing the binding of 14–3-3 proteins [29, 32].

### PIPKs

PIP_2_ can be generated by two different biosynthetic pathways. In the canonical route for synthesis of PIP_2_, a second phosphorylation of PI 4-phosphate (PI4P) at position 5 hydroxyl residue is catalyzed by type I PIPKs (PIPKIs) to generate PIP_2_ [33]. In the non-canonical route, PI 5-phosphate (PI5P) can be phosphorylated by type II PIPKs (PIPKIIs) at position 4 hydroxyl residue to generate same PIP_2_ as well [34]. PI5P is relatively low-abundance and this PIPKII mediated phosphorylation is considered to be a mechanism to regulate its levels in the cell. The Type I PIPKs have three distinct isozymes termed as PIPKIα (*PIP5K1A*), PIPKIβ (*PIP5K1B*), and PIPKIγ (*PIP5K1C*) [33]. The mRNA coding for PIPKIγ can be alternatively spliced to give rise to PIPKI γ635, γ661 and γ687 variants [35]. PIPKIIs are PIPKIIα (*PIP4K2A*), PIPKIIβ (*PIP4K2B*), and PIPKIIγ (*PIP4K2C*) isozymes [36]. Structurally, both types of PIPKs share a homologous central lipid kinase domain (PIPKc) and a conserved dimerization domain. The PIPKc has an activation loop at C-terminal that is specific for substrate and subcellular localization. The variability on N- and C-terminal also facilitates isoform-specific functions of PIPKs [37] (Figure 2B).

Different homo-dimerization mechanisms have been reported for PIPKIs and PIPKIIs in animal models, giving rise to the idea of different interaction surfaces for their binding partners. The PIPKIs have been reported to be activated by Ras homolog family member A (RhoA)/Rac family small GTPase 1 (Rac1), ADP ribosylation factor 6 (ARF6) GTPases, and Wnt signaling through dishevelled segment polarity protein (DVL), in an isoform-selective manner [38–40]. PIP5KII’s isoforms, on the other hand, can form both homo- and heterodimers which may modulate its activity. The activity of PIPKIIα is modulated by phosphorylation of its activation loop by protein kinase D, while the activity of PIPKIIβ is regulated by p38 mitogen activated protein kinase (MAP kinase) [41–43]. PIPKIIβ is also proposed as a GTP sensor in cells, because of its preference for GTP instead of ATP [44]. Not much is known when it comes to regulation of PIPKIIγ activity, due to it being non-functional when expressed in bacterial cells [45]. Both types of PIPKs play important role in development, actin dynamics, autophagy, and polarity of cells [17, 46]. A type III class of PIPKs (PIPKIII, or PIKfyve) also exists which is involved in phosphorylating PI and PI3P at position 5 hydroxyl residue to produce PI5P and PI(3,5)P_2_ respectively, but its characterization is beyond the scope of this review.

### PI3K

The PI3Ks are divided into three classes based on their structure and substrate specificity. Class I PI3K catalyzes phosphorylation of position 3 hydroxyl residue of PIP_2_ to generate PIP_3_. Based on their structure and composition, Class I PI3Ks are further divided into two subclasses i.e., Class IA PI3Ks and Class IB PI3Ks. Class IA PI3Ks are heterodimeric proteins consisting of a catalytic subunit p110α, p110β, or p110δ; encoded by *PIK3CA/B/D* genes and a regulatory subunit [p85/55/50α, p85β, or p55γ variants; encoded by *PIK3R1/2/3* genes respectively) [47, 48]. These regulatory subunits play important role in stabilizing the catalytic subunits, inhibiting their activity, and recognize the phosphorylated YXXM motif on intracellular receptors and adaptors through their Src homology 2 (SH2) domains to allow for plasma membrane localization, adaptor binding and increased kinase activity [49, 50]. Class IB PI3Ks consist of a p110γ (*PIK3CG*) catalytic subunit, which can associate with either p101 (*PIK3R5*), or p84 (also known as p87; *PIK3R6*) regulatory subunits to form a heterodimer that can get activated through interaction with Gβγ subunits of GTP-binding proteins [51]. In fact, both subclasses can be activated through interaction of GTP-Ras at the Ras-binding domain. The C2 and helical domains are thought to take part in membrane tethering [52] (Figure 2C).

The ability to generate PIP_3_ depends on the tissue specific expression and activation of the specific isoforms by the RTKs and GPCRs. There is considerable overlap in RTKs and GPCRs mediated downstream signaling, leading to synergistic activation of different PI3K isoforms [53]. In the context of neurons, the p110α subunit is mainly activated through RTKs and plays important role in insulin signaling which is important for cell survival, energy metabolism, synaptic development, and plasticity [54–56]. The p110β subunit is primarily associated with GPCRs and is reported to regulate glutamate receptor dependent (mGlu1/5) form of plasticity and protein synthesis in the brain [57, 58]. The P110γ has been implicated in *N*-methyl-*D*-aspartate (NMDA)-dependent neuronal plasticity, while P110δ seems to be involved in axonal growth and regeneration [59, 60].

### Phosphatases

Phosphoinositide phosphatases are enzymes that catalyze removal of phosphate groups from the position 3,4 or 5 hydroxyl residues of myo-inositol ring of PIs. Just like PI kinases, these phosphatases also have substrate and catalytic site specificity. Here, phosphatases that recognize only PIP_2_ or PIP_3_ as their substrates are in focus.

### Phosphoinositide 3-phosphatases

**Figure 3.**
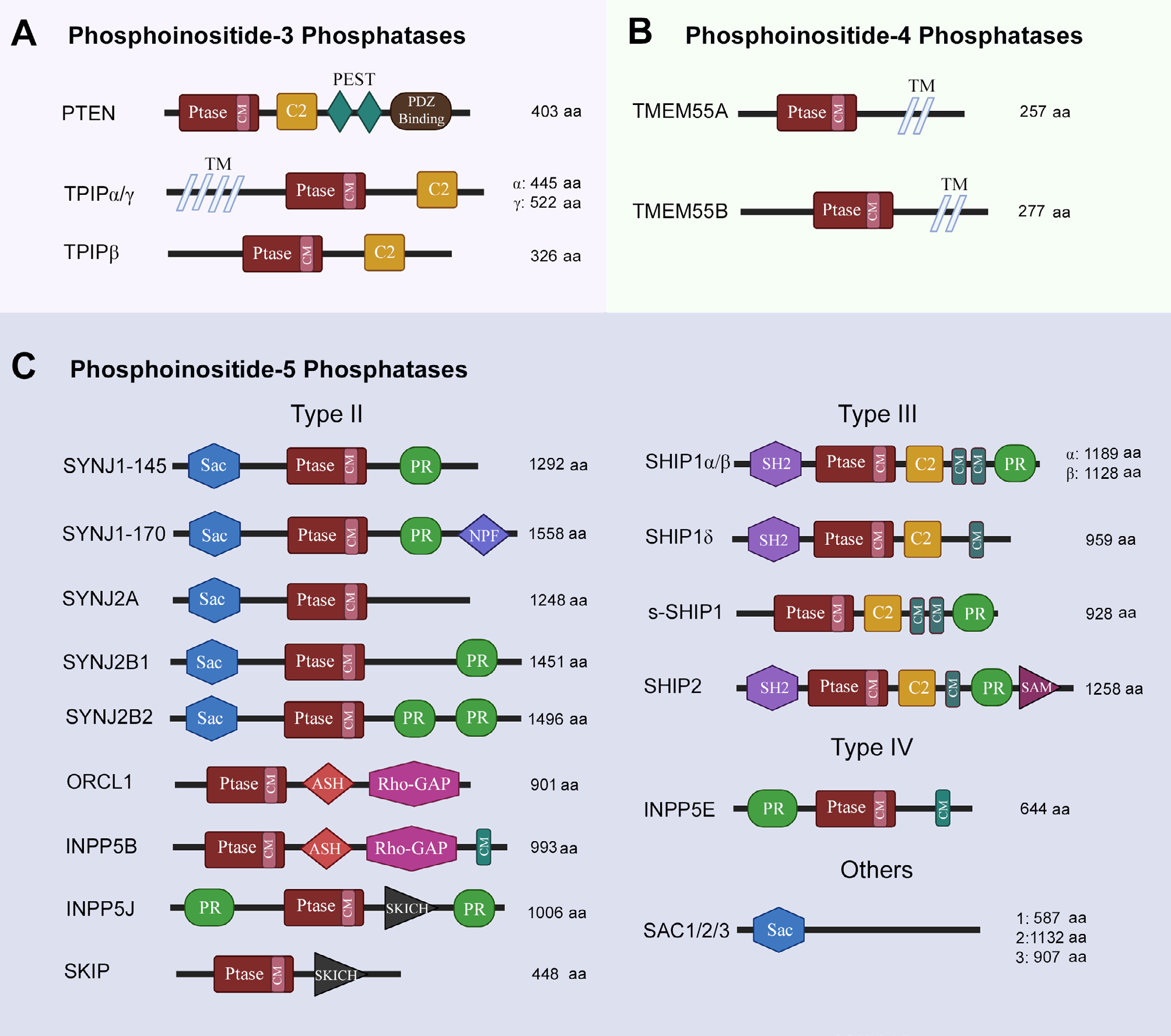
Structural features of phosphoinositide phosphatases. Ptase: phosphatase domain; CM: consensus motif; PEST: proline, glutamine, serine, threonine-rich sequence; TM: transmembrane spanning region; NPF: asparagine, proline, phenylalanine repeats; SKICH: skeletal muscle- and kidney-enriched inositol polyphosphate phosphatase (SKIP) carboxyl hydroxy domain; SH2: Src homology 2 domain; SAM: sterile α motif; SYNJ: synaptojanin; ORCL1: oculocerebrorenal syndrome of Lowe-1; ASH: ASPM-SPD-2-Hydin; Rho-GAP: Rho GTPase activating protein; SHIP: SH2 containing inositol phosphatase; s-SHIP: stem cell specific isoform of SHIP. The schematics for structural domains are not to scale

Phosphoinositide 3-phosphatases (PI3 phosphatases) primarily dephosphorylate PIP_3_ on position 3, thereby antagonizing the activity of Class I PI3 kinases and generating PIP_2_. The activity of these phosphatases is important for maintaining a PIP_2_/PIP_3_ balance and even small changes may have dramatic effects on neuronal growth and development. The PI3 phosphatases include PTEN, TPTE, and TPIP. PTEN is a dual-specificity lipid and protein phosphatase. Structurally, PTEN contains a CX5R catalytic motif in its phosphatase domain which is common to all PI3 phosphatases; a lipid-binding C2 domain which mediates membrane binding; two C-terminal PEST (proline, glutamine, serine, threonine) sequences that enhance sensitivity to proteolysis; and a PDZ domain important for stability and binding proteins [61] (Figure 3A). PTEN is distributed throughout the cell but has the highest catalytic activity when associated with membranes [62]. Nuclear-associated PTEN has tumor-suppressor activity [63], while nuclear-excluded PTEN has been associated with dysregulation of neuronal growth [64]. *PTEN* gene activity is reported to be modulated by its splice variants and post transcriptional modifications [65]. Whether PTEN can access nuclear phospholipids is currently a debated topic in the literature.

TPTE (*TPTE*) is found in plasma membrane but is reported to lack PI phosphatase activity [66]. TPIP (*TPTE2*) exists as three isoforms (TPIPα, β and γ). TPIPα is endoplasmic reticulum (ER)-localized while TPIPγ is reported to be cytosolic [67]. All isoforms have a phosphatase domain and a C2 domain. TPIPα and TPIPγ are predicted to have transmembrane (TM) segments that display homology to voltage-sensing phosphatases (VSP) [67, 68]. The best characterized VSP is found in marine invertebrate *Ciona intestinalis*, which contains a voltage sensing domain (VSD). This ci-VSP recognizes both PIP_3_ and PIP_2_ as its substrate for dephosphorylation and gets activated in response to membrane depolarization [69].

### Phosphoinositide 4-phosphatases

Phosphoinositide 4-phosphatases (PI4 phosphatases) catalyze the removal of phosphorylation from position 4 of myo-inositol has group of PIs. Two types of PI4 phosphatases have been identified in mammals, namely INPP4 and TMEM55. Out of these two, only TMEM55 proteins (TMEM55A, TMEM55B; encoded by *TMEM55A/B*) will be discussed as they recognize PIP_2_ as their substrate to dephosphorylate position 4, converting it into PI5P [17]. Both TMEM55A and TMEM55B proteins are expressed ubiquitously; contain a CX5R motif in their phosphatase domain, and are named after two TM segments on their C-terminal [70] (Figure 3B). TMEM55 proteins are not well-characterized but have been reported to be involved in embryonic growth factor receptor (EGFR) degradation, cholesterol homeostasis, DNA damage response and p53-mediated cell death [70–73].

### Phosphoinositide 5-phosphatases

Phosphoinositide 5-phosphatases (PI5 phosphatases) are the most abundant lipid phosphatases. There are three classes (II, III, and IV) of INPP5s with an inositol 5-phosphatase (5-Ptase) domain, which contains motifs for phosphoinositide substrate selectivity [74, 75]. The type II enzymes are synaptojanins, ORCL1, INPP5B, INPP5J, and SKIP. Both synaptojanin isozymes (*SYNJ1* and *SYNJ2*), including their splice variants (145, 170, A, B1, B2), share similar structures consisting of a N-terminal Sac domain, a central 5-Ptase domain and a C-terminal PR region. All synaptojanins dephosphorylate PIP_2_ and PIP_3_ on position 5 [76, 77]. Both SYNJ1–145 and SYNJ2B are reported to be especially localized in nerve terminals and synapsis [78, 79]. The ORCL1 (*ORCL*) also removes the 5-phosphate from PIP_2_ and PIP_3_; contains 5-Ptase, ASH and Rho-GAP-like domains; but only one of its splice variants (ORCL1a) is expressed in brain [80–82]. INPP5B (*INPP5B*) is structurally similar to ORCL, but with an additional CAAX motif on C-terminal [83]. INPP5B expression has not been reported in brain. INPP5J (*INPP5J*), on the other hand, has been detected in brain; contains an additional SKICH domain; and seems to be involved in neurite elongation [84–86]. SKIP (*SKIP*) itself has a preference for PIP_3_ as a substrate and is expressed ubiquitously [87].

SHIP family enzymes (*SHIP1* and *SHIP2*) are type III PI5 phosphatases whose alternative splicing gives rise to SHIP1α, SHIP1β, SHIP1γ and s-SHIP1 [88]. All SHIP family enzymes contain an SH2 domain, a PR region, and a NPXY motif whose phosphorylation allows for interactions with binding partners containing immunoreceptor tyrosine inhibitory motif (ITIM)/ immunoreceptor tyrosine activating motif (ITAM), phosphotyrosine-binding (PTB) or SH2 domains. SHIP2 also has an additional SAM domain [17, 89]. All SHIP1 isoforms and SHIP2 recognize PIP_3_ as their substrate, however, only SHIP2 is expressed ubiquitously [90]. Pharbin (*INPP5E*) is the only type IV PI5 phosphatase, contains a PR region, 5-Ptase domain and a CAAX motif; is expressed in brain; and has the highest affinity (*K*_m_ = 0.65 μmol/L for PIP_3_ recognition as a substrate than any other PI5 Phosphatase [91]. Another family of PI5 phosphatases is Sac family of phosphatases (Sac1, Sac2, Sac3), which differ from the rest by the lack of 5-Ptase domain. Sac2 and Sac3 dephosphorylate both PIP_2_ and PIP_3_ [92] (Figure 3C). Sac2 and Sac3 are expressed ubiquitously but Sac2 expression is especially high in the brain tissue. Both Sac2 and Sac3 have been implicated in neuronal outgrowth [93, 94].

### Phospholipases

**Figure 4.**
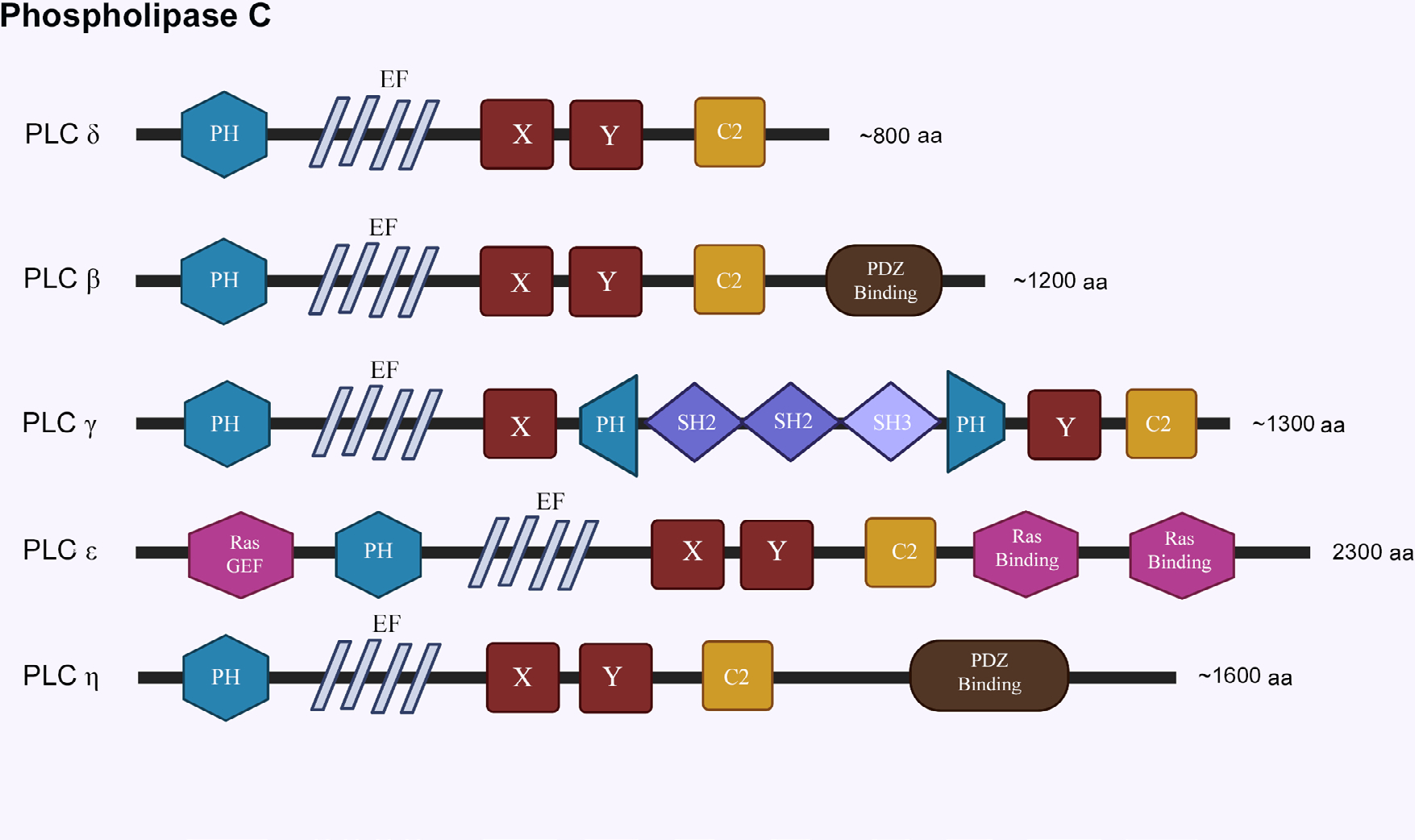
Structural features of phospholipase C family. EF: helix-loop-helix motif; X,Y: TIM barrel domain; SH2: Src homology 2 domain; SH3: Src homology 3 domain. The schematics for structural domains are not to scale

Phospholipases are enzymes that hydrolyze phospholipids into its constituent fatty acids. The most relevant class of phospholipases to our discussion is phospholipase C (PLC) class of PI-specific enzymes, which cleave PIP_2_ to generate diacylglycerol (DAG) and inositol-1,4,5-triphosphate (IP_3_). DAG and IP_3_ are important secondary messengers that are involved in protein kinase C (PKC) signaling, intra-neuronal calcium (Ca^2+^) signaling, and transcription, among other regulatory roles [6]. PI specific PLCs have been classified into six families (β, γ, δ, ε, ζ, and η; Figure 4). Alternative splicing reportedly leads to the generation of about 30 isozymes in mammals [95]. All PI specific isozymes have a homologues core consisting of a N-terminal PH domain, four EF domains, a triose phosphate isomerase (TIM) barrel domain (X, Y), and a C-terminal C2 domain [96, 97]. The subtle structural nuances and specific combinations of these domains can regulate distribution and function of each PLC isozyme. PLC enzymes are activated through either GPCRs (β, δ, and η), or RTKs (γ and ζ), or both (ε) [98]. Of note here is a suggested role for the nuclear PLC-β1 in PIP_2_ hydrolysis, raising questions about the existence of PI signaling in the nucleus [99]. The isoforms PLC-δ (1,3,4), PLC-β (1,4), PLC-γ (1), PLC-ε and PLC-η (1,2) are most relevant to nervous system and have been implicated in neurodegenerative disorders [100].

### Cholesterol-rich microdomains

The cellular plasma membranes are heterogenous in nature and are organized into microdomains or ordered/disordered liquid phases, based on their lipid and protein constituents. In the last decade or so, it has been reported that composition of these microdomains dictates localization of PIs to specific regions of the plasma membranes [101]. An important regulator of this localization is reported to be cholesterol. Plasma membrane microdomains can be classified, on the basis of cholesterol abundance, into cholesterol-rich, liquid-ordered (L_o_) raft domains or cholesterol-poor, liquid-disordered (L_d_) non-raft domains. Recent literature suggests that PIP_2_ is present in both domains, however, it gets hydrolyzed by PLC faster, and is also restored more rapidly in cholesterol-rich (L_o_) domain [102]. This compartmentalization of PIP_2_ signaling seems to be conserved as it has been reported in plants membranes as well [103]. In the context of neurodegenerative diseases, increasing cholesterol levels in the membranes led to PLC-mediated depletion of PIP_2_ and an increase in AD-associated secretory amyloid β42 in cell lines [104]. PTEN phosphatase binding to PIP_2_ is also reported to be increased in cholesterol-rich environments [105].

## Effectors of PIP_2_ and PIP_3_ signaling

Classically, PIP_2_ and PIP_3_ were primarily thought of as precursors to secondary messengers that mediate activity of integral cellular signaling pathways through interaction with cytoplasmic proteins. However, PIP_2_ and PIP_3_ also play direct roles in mediating the activities of membrane-bound ion channels and transporters. These direct and indirect roles of PIP_2_ and PIP_3_ signaling in mediating cellular development and function are described below:

### IP_3_ and DAG signaling

GPCRs or RTKs mediated activation of PLC isoforms leads to hydrolysis of PIP_2_ into IP_3_ and DAG [6]. IP_3_ binds to IP_3_ receptors (IP_3_Rs) on the ER to release Ca^2+^. These IP_3_Rs are found on nuclear envelope as well. In order for Ca^2+^ to release from ER into the cytosol, IP_3_ first needs to bind to all four monomers of an IP_3_R tetramer, causing a conformational change that allows Ca^2+^ to pass. This allowance is, however, transient. When Ca^2+^ levels rise above a certain level, this signaling becomes inhibitory through complex feedback interactions [106–108]. These IP_3_ induced Ca^2+^ oscillations are found in multiple cell types. In brains, these oscillations regulate differentiation and proliferation [109]. IP_3_ levels regulate steering of axonal growth cones, while Ca^2+^ transients specify if a neuron will be inhibitory or regulatory by way of regulating neurotransmitters release [110]. Low-frequency oscillations lead to release of excitatory neurotransmitters (acetylcholine, glutamate), while higher frequency oscillations lead to expression of inhibitory transmitters (glycine, γ-aminobutyric acid) [111]. These oscillations are important in generating brain rhythms for sleep/wake cycle, memory formation, and synaptic plasticity [112, 113]. Alterations in IP_3_/Ca^2+^ signaling are associated with many neurological disorders like AD, ASD, epilepsy, schizophrenia among others, which are discussed in section Disease relevance.

**Figure 5.**
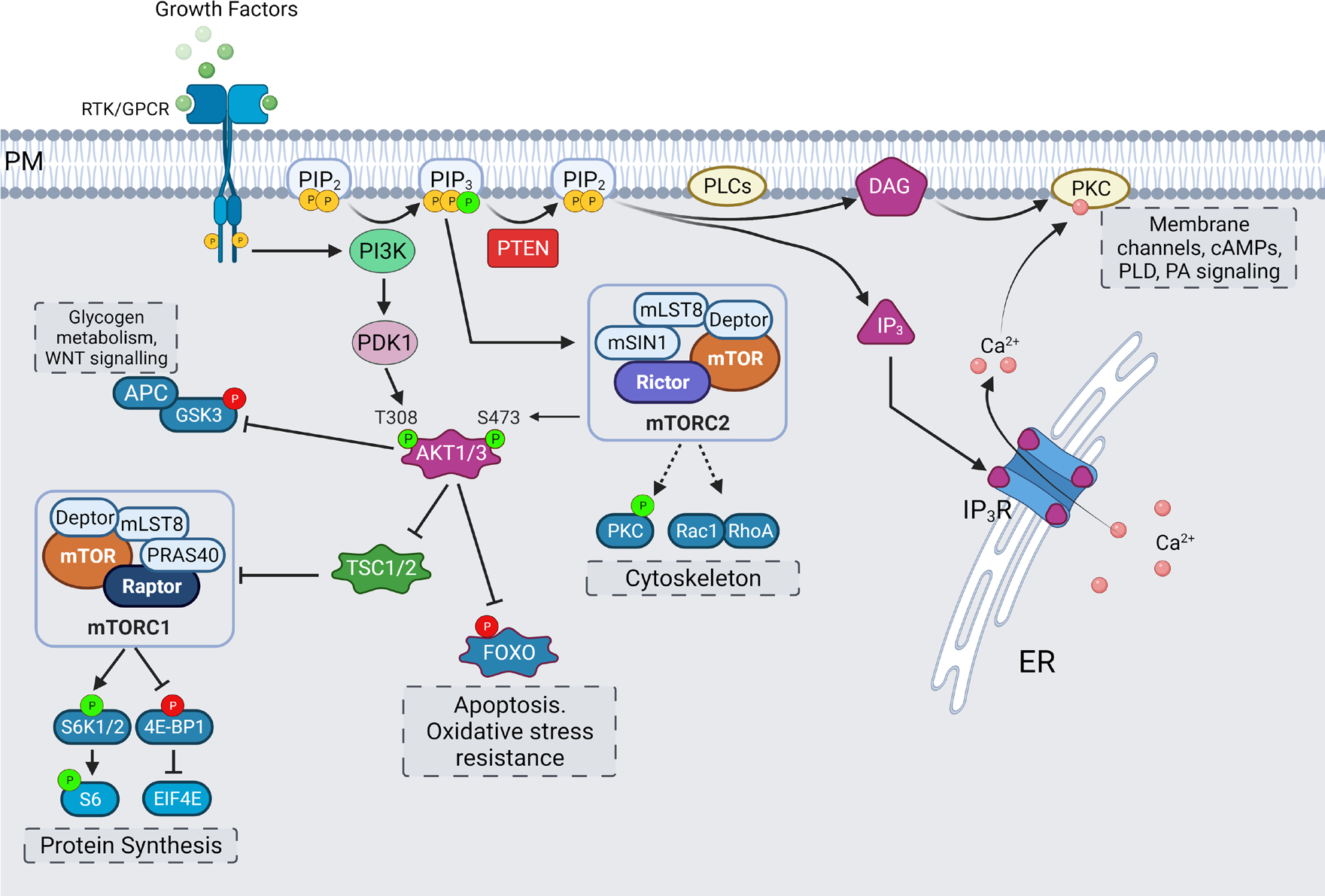
Effectors of PIP_2_ and PIP_3_. The hydrolysis of PIP_2_ by PLC leads to formation of IP_3_ and DAG which are involved in intracellular calcium release and activation of PKC-mediated signaling. PI3K-mediated synthesis of PIP_3_ leads to activation of AKT/mammalian target of rapamycin (mTOR) signaling pathway, with mTOR complexes, glycogen synthase kinase 3 (GSK3)β and Forkhead BOX O (FOXO) working as main effectors for regulation of processes of protein synthesis, cytoskeletal organization, and nutrient-sensing and survival. PTEN phosphatase keeps this activation in balance by dephosphorylation of PIP_3_ into PIP_2_. Activating phosphorylations are shown in green, while inhibitory phosphorylations are shown in red. 4E-BP1: eukaryotic initiation factor 4E-binding protein 1; EIF4E: eukaryotic translation initiation factor 4E; APC: adenomatous polyposis coli; Deptor: DEP domain-containing mTOR-interacting protein; mLST8: mammalian lethal with sec13 protein 8; mSIN1: mammalian stress activated protein kinase interacting protein; mTORC1: mammalian target of rapamycin complex 1; PDK: phosphoinositide-dependent protein kinase; PM: plasma membrane; PRAS40: proline-rich Akt substrate of 40 kDa; Raptor: regulatory associated protein of mTOR; Rheb: Ras homolog enriched in brain; Rictor: rapamycin-insensitive companion of mTOR; S6: ribosomal protein S6; S6K: ribosomal S6 kinase; TSC: tuberous sclerosis complex

DAG, the second product of PIP_2_ hydrolysis, is most notably involved in PKC signaling. The PKC family of kinases have two DAG-binding copies of C1 domains, and in the case of conventional PKCs (cPKCs), an additional C2 domain to sense intracellular Ca^2+^ levels for full activation [114, 115]. The subcellular distribution of cPKCs is cytosolic under basal Ca^2+^ conditions, and it has now been shown that Ca^2+^ binding to C2 domain is sufficient and necessary for rapid membrane translocation. The DAG association with C1 domain of cPKC is important for its retention on the membrane [116]. Hence, the downstream signaling of both PIP_2_ hydrolysis products is intricately connected and has critical roles to play in activation of cPKCs. The activation of cPKCs has been implicated in interfering with inhibitory binding of calmodulin at plasma membrane Ca^2+^-ATPases (PMCA); regulation of transient receptor potential (TRP), Na^+^/Ca^2+^ exchanger (NCX) and sodium proton exchangers (NHE) channels; generation of cyclic adenosine monophosphates (cAMPs); and activation of phospholipase D (PLD) and DAG kinases to coordinate phosphatidic acid (PA) signaling [117–121] (Figure 5). The PA signaling is known to play role in neurite growth associated cytoskeletal and membrane remodeling [122]. Alterations in PA signaling levels are associated with glioblastomas, intellectual disability, and neurodegenerative disorders, which are discussed later.

### AKT and mTOR pathway

Canonically, the activation of RTKs or GPCRs by extracellular stimuli recruits PI3K to plasma membrane, where it catalyzes the phosphorylation of PIP_2_, which generates PIP_3_. This generation of PIP_3_ leads to activation of AKT (also known as protein kinase B)/mTOR signaling pathway, which is a central regulator for cell growth, metabolism, protein translation, cytoskeletal organization, membrane trafficking and survival [123]. PTEN is a negative regulator of this pathway which keeps the activation of this pathway in check, by dephosphorylating PIP_3_ into PIP_2_ [124]. The formation of PIP_3_ recruits PH domain containing proteins like PDK and AKT to the plasma membrane [125]. The PDK is reported to localize in cholesterol-rich membrane rafts [126]. This close proximity allows PDK to phosphorylate AKT at T308 [127]. Phosphorylation at T308 stabilizes and activates AKT.

For AKT to reach its maximal activity potential a further phosphorylation at S473 is needed [128, 129]. It is thought that first phosphorylation event at T308 primes AKT for second phosphorylation at S473, which in turn, stabilizes the first T308 phosphorylation. This idea is supported by the observations that T308 phosphorylation can occur without prior S473 phosphorylation, but not vice versa [130]. The S473 phosphorylation is brought about by mTOR complex 2 (mTORC2). This complex is assembled by PIP_3_ binding to the PH-domain containing mSIN1, and thereby relieving its suppression on mTOR kinase activity and localizing it to plasma membrane as well [131]. mTORC2 complex also contains a scaffolding protein Rictor which regulates its assembly. mTORC2, as the name of its scaffolding protein suggests, is insensitive to acute rapamycin treatment and is known to regulate cytoskeletal rearrangements to support formation of new dendritic branches [129, 132].

Targeting Rictor to inhibit mTORC2 activity has been shown to result in inhibition of basal synaptic transmission and dendritic outgrowth in hippocampal neurons [133]. Another candidate for mediating this S473 phosphorylation on AKT is DNA-dependent protein kinase catalytic subunit (DNA-PKcs), but its role has been described mostly in the context of DNA damage in the nucleus [134]. Reports of PIP_3_ being enriched at endosome and nuclear envelope also support the idea of localized phosphorylation and activation of AKT in intracellular compartments [135, 136]. In addition, three subtypes of AKT (AKT1, AKT2, AKT3) exist, with a high degree of homology in amino acid sequence and corresponding phosphorylation sites. Mostly, only AKT1 and AKT3 expression has been reported in hippocampus, while AKT2 expression is limited to cerebellum [137].

AKT targets many substrates which are at the nodes of different signaling pathways. One such target protein is GSK3 whose inhibitory phosphorylations of its targets are relieved by an inactivating phosphorylation by AKT. GSK3 regulates cell growth and development by regulating glycogen metabolism. It is also involved in Wnt/β-catenin signaling pathway, indicating its role in crosstalk between these two pathways [138, 139]. Another downstream effect of AKT activation is the assembly of mTORC1. mTORC1 is not a direct substrate of AKT. AKT mediated inhibitory phosphorylation on TSC relieves its inhibition of Rheb-GTP formation and leads to subsequent activation of mTORC1. In addition, mTORC1 complex assembly is facilitated by a scaffolding protein Raptor, whose suppression is relieved by AKT-mediated phosphorylation of PRAS40 [140–142] (Figure 5).

Broadly, mTORC1 is involved in processes of enhanced protein synthesis and growth through downstream effectors like S6 kinase; lipid synthesis through sterol responsive element binding protein (SRBEP); cellular stress responses through its negative regulator AMPK; and cell survival through autophagy and ubiquitin proteosome regulation [143–146]. In neuronal context, mTORC1 is activated through stimuli like brain-derived neurotrophic factor (BDNF), reelin, glutamate, gamma-aminobutyric acid (GABA), acetyl choline, and neuropeptides [147–149]. The stimulation of mTOR increases dendritic protein synthesis locally and contributes to synaptic and structural plasticity [150, 151]. mTOR activity is also needed for proper dendritic arborization, axonal branching, neuronal polarization, and autophagy mediated differentiation [152–154].

AKT also targets transcription factors of FOXO family. These transcription factors translocate from cytoplasm to nucleus to regulate processes like apoptosis and oxidative stress resistance. In the nervous system, acute FOXO activity is involved in age-dependent axonal degeneration, spine density and consolidation of memories [155, 156].

### Ion transporters and channels

Apart from signaling through its downstream effectors, PIP_2_ may directly bind to and interact with ion transporters and membrane channels to affect their activity [11]. The phospholipid composition of the intracellular compartments has been hypothesized to regulate the activity of membrane channels in space, until they arrive at their target membrane compartment with their signature phospholipid composition. Another hypothesis is about regulation of channels’ activity in time. Cells may respond dynamically to extracellular stimuli by way of changes in PI signaling mediating cell’s electrical and transport activity [157, 158]. This is an area of active investigation and the exact nature of PI binding to membrane channels has been difficult to determine. However, recent crystallography and mutagenesis studies have identified clusters of conserved amino acid residues on the channels that interact with phosphate group of PIs. These interactions might cause conformational changes to stabilize channel protein in a certain state, as observed with crystal structures of PIP_2_ bound to potassium channels. The conformational change brought about by binding of PIP_2_ is thought to lead to channel activation [159, 160].

In the context of potassium channels activity, PIP_2_ seems to be the principal phospholipid regulating their activity. For voltage-gated potassium channels (Kv), the Kv7 or potassium voltage-gated channel subfamily Q (KCNQ) family is most relevant to neuronal excitability [161]. The PLC mediate depletion of PIP_2_ reduces their current in a matter of seconds [159]. Direct application of PIP_2_ experimentally, slows this rundown [160]. Structurally, basic residues on the C-terminal TM segment and calmodulin binding may be required for PIP_2_ coupling to KCNQ channels [162]. Inwardly rectifying potassium channels (Kir) were the first to be identified as PIP_2_ dependent [163]. All members of this family bind to PIP_2_, but do so with different affinities (high affinity: Kir1, 2.1 and 4; low affinity: Kir2.3 and 3). High affinity channels were slowest to run down in response to PIP_2_ depletion [164]. PIP_2_ stabilizes the open state of these channels by binding to basic and hydrophobic residues in the N- and distal C-terminal [165].

Calcium activated potassium channels have also been shown to regulated by PIP_2_ levels in the cell [166, 167]. For voltage-gated calcium channels (CaV), PIP_2_ is required for opening in response to membrane potential changes. PIP_2_ is hypothesized to bind to CaV as a segmented ligand bringing two parts together, and is competed for interaction by CaV β subunits whose expression governs the PIP_2_ sensitivity of these channels [168]. Some of the TRP channels are also PIP_2_ sensitive, i.e., PIP_2_ promotes their opening by binding to basic residues on cytoplasmic linker and C-terminal [169, 170]. An example being cold-activated TRPM8 channel is that it does not respond to stimuli in the absence of PIP_2_ [171]. The P2X receptor, CNG, and TMEM16-ANO1 are examples of some other channels that are also sensitive to PIP_2_ binding [158].

### Actin regulatory proteins

Actin provides an architectural scaffold for the cell, and is an important regulator of cellular shape, trafficking, and migration [172]. This actin network is integral to the morphological remodeling of highly specialized cells like neurons. PIs regulate the activity of actin-binding proteins which are reported to control the initiation of processes like spinogenesis and dendritogenesis.

In general, PIP_2_ inactivates actin-biding proteins that inhibit actin polymerization, while activating proteins that support polymerization. Gelsolin is an actin severing protein, whose binding with PIP_2_ frees up the ends of actin filament for polymerization [173]. Interestingly, this binding is enhanced by calcium ions to promote actin polymerization [174]. The actin-related protein 2/3 (Arp2/3) complex which is responsible for branching of actin filaments is also activated through binding of PIP_2_ with its activation protein, Wiskott-Aldrich syndrome protein (WASP) [175]. PIP_2_ has also been shown to bind to other actin regulatory proteins like cofilin and profilin. The severing action of cofilin is inhibited by binding with PIP_2_, while PIP_2_-profilin interaction inhibits PLCγ-mediated hydrolysis of PIP_2_ [176, 177].

The neuronal membrane branching/bending into dendrites, axons or spines is observed to protrude from specialized filopodia like structures which are filled with actin. PIP_2_ is reported to mediate the formation of these filopodia. PIP_2_ recruits the inverse bin-amphiphysin-rvs (I-BAR) protein missing-in-metastasis (MIM)/MTSS1 and Arp2/3-mediated actin assembly to nucleate the formation of protrusion that are pre-cursors to spinogenesis [178]. Nerve growth factor (NGF) treatment is known to increase the formation of patches that are precursors to the formation of axonal filopodia in a PI3K mediated activity dependent manner [179]. Localized microdomains of PIP_3_ are also reported to be synchronous with formation of these precursor patches that are formed in response to NGF treatment [180]. Application of brain derived neurotrophic factor was reported to enhance PIP_3_ localization to dendritic filopodia as well [181].

## Disease relevance

The intricate balance between the levels of PIP_2_ and PIP_3_ is necessary for proper regulation and maintenance of several critical cellular processes in the nervous system. Any perturbation to this balance, be it through kinases or phosphatases, may lead to deficits in brain development and neuronal regulation, manifesting itself in disease and disorders of the nervous system.

### Disorders of neurodevelopment

Mutations in genes encoding for proteins involved in PI synthesis and metabolism have been associated with ASD. Of the kinases involved in PIP_2_ and PIP_3_ synthesis, mutations in catalytic and regulatory isoforms of PI3K have been observed to be overrepresented when it comes to disorders affecting brain development [15]. In the context of dysregulation of catalytic subunits of PI3K, mutations in *PIK3CA* gene have been observed in clinical cases of cortical dysplasia and megalencephaly [182]. Another study described missense mutations in gene coding for p110α in a patient with autism and macrocephaly [183]. There is also strong evidence for overexpression of p110β catalytic subunit in some cases of autism [184]. This overexpression has been shown to be caused by chromosomal duplication.

More studies have linked a loss of Fragile X mental retardation protein (FMRP) in Fragile X syndrome (FXS) to p110β overexpression [185]. Since p110β mRNA binds to FMRP, this loss of FMRP is associated with increase in p110β expression. This increase has been observed in mouse models [185], as well as human patients cell lines [186, 187]. Dysregulation of another p110δ subunit of PI3K has also been observed in autism and schizophrenia [188, 189]. The location for gene coding for p110γ has been identified as a potential autism susceptible locus [190]. As for the PI3K regulatory subunits, mutations in p85β have been described to be associated with autism and megalencephaly [191]. So far, members of other classes of PI3K (class II and III) that are involved in generation of PIs other than PIP_2_ and PIP_3_ have not been shown to be strongly associated with the incidence of autism, further highlighting the importance of maintaining PIP_2_ an PIP_3_ balance for neuronal health.

In addition to PI3K, PI4K and PIPKs have also been shown to be altered in autism and other related disorders. Deleterious mutations in a regulator of PI4K have been identified in patients with autism [192]. PIPK isoform 3 has been found to be duplicated in patients with developmental delay and autism [193]. Among the phosphatases regulating PIP_2_ and PIP_3_ balance, PTEN stands out for its role in developmental delay, autism, and epilepsy [194–196]. In multiple animal model studies, PTEN loss has been shown to cause autism-like phenotypes and behavior [197–199]. PTEN loss associated phenotypes include anxiety, seizures, macrocephaly, and deficits in neuronal migration, growth, electrical activity, and social behavior [197, 199–201].

Trisomy of the locus containing SYNJ1 phosphatase has been shown to result in enlarged endosomes in cell lines developed from patients with Down’s syndrome (DS) [202]. Other PI phosphatases have not been linked with autism but have been associated with neurodegeneration.

### Disorders of neurodegeneration

The PIP_2_/PIP_3_ balance in the intracellular environment is reported to be perturbed in neurodegenerative disorders like Alzheimer’s and Parkinson’s [203]. SYNJ1, a PI phosphatase regulating synaptic activity, was observed to be increased in autopsy of adult brains of DS and early-onset AD patients [204]. The excess of SYNJ1 was also found to contribute to memory deficits in mouse models of AD. Reducing this excess experimentally was found to accelerate clearance of Amyloid β (Aβ) and associated cognitive decline. In another study, restoring PIP_2_ levels was sufficient to ameliorate such synaptic dysfunction [205, 206]. Presence of tau protein has also been detected in patients with SYNJ1 mutations [207]. Missense mutation in sac1 domain of SYNJ1 has also been found in patients with early onset PD [208]. These patients experienced tremors and some cortical atrophy as well. Loss-of-function mutations in SYNJ1 have been observed in patients with infantile epileptic encephalopathy [209, 210].

Single nucleotide polymorphism (SNP) in INPP5B phosphatase have been shown to be associated with sporadic amyotrophic lateral sclerosis (ALS) [211]. Loss of function mutations in chorein or vacuolar protein sorting-associated protein 13A (*VPS13A*) have been found in patients with a rare hereditary genetic disorder called chorea-acanthocytosis (ChAc) [212]. ChAc is characterized by progressive movement disorder, seizures, cognitive difficulties, and neurodegeneration-particularly in striatum [213–215]. The mechanism behind disease pathology has remained puzzling for a while. However, recent studies in neuronal cell cultures and animal models have hinted at a role for PI signaling in the development of ChAc. Chorein is reported to be involved in activation of p85 regulatory subunit of PI3K, with subsequent activation of several downstream kinases [215, 216]. Compromised cytoskeleton and cell-survival was observed in patient-derived neuronal cell cultures containing mutations in chorein [217]. Mutations in genes encoding for IP_3_R, for example inositol 1,4,5-trisphosphate receptor type 1 (*ITPR1*), have been reported in infantile-onset nonprogressive spinocerebellar ataxia (SCA) [218]. SCA are degenerative disorders related to movement control. The dysfunction of IP_3_R leads to aberrant calcium signaling in cerebellar neurons which are implicated in SCA pathogenesis [219]. Mutations in *FAM126A* gene leading to loss of hyccin/FAM126A protein, a scaffolding partner of PIKIIIα, causes disorders of progressive hypomyelination in central and peripheral nervous system [30]. This hypomyelination manifests in form of leukoencephalopathy known as hypomyelination and congenital cataract (HCC), with symptoms of cognitive deficits and neuropathy [220].

## Conclusion

Phosphoinositide signaling, especially the levels of PIP_2_ and PIP_3_, is critically regulated for maintenance of general neuronal health by varied kinases and phosphatases. The downstream functions for PIP_2_ and PIP_3_ are quite diverse, and include roles in neuronal growth, development, connectivity, and activity. Perturbations to the regulators of this balance in PIP_2_ and PIP_3_ have been associated with myriad of neurological disorders and diseases. Research in this domain continues and new associations are being discovered regularly. However, the need for effective therapeutic strategies remains.

## Abbreviations

5-Ptase: inositol 5-phosphatase
ACBD3: acyl-CoA-binding protein 3
AD: Alzheimer’s disease
ASD: autism spectrum disorder
CaV: voltage-gated calcium channels
ChAc: chorea-acanthocytosis
cPKC: conventional protein kinase C
DAG: diacylglycerol
ER: endoplasmic reticulum
FMRP: Fragile X mental retardation protein
FOXO: Forkhead BOX O
GPCR: G-protein coupled receptor
GSK3: glycogen synthase kinase 3
INPP5: inositol polyphosphate 5-phosphatase
IP_3_: inositol-1,4,5-triphosphate
IP_3_R: inositol-1,4,5-triphosphate receptor
Kir: inwardly rectifying potassium channels
mTORC1: mammalian target of rapamycin complex 1
mTORC2: mammalian target of rapamycin complex 2
ORCL: oculocerebrorenal syndrome of Lowe
PA: phosphatidic acid
PD: Parkinson’s disease
PDK: phosphoinositide-dependent protein kinase
PH: pleckstrin homology
PI: phosphoinositides
PI3K: phosphoinositide 3-kinase
PI4K: phosphatidylinositol-4 kinases
PI5 phosphatases: phosphoinositide 5-phosphatases
PI5P: phosphatidylinositol 5-phosphate
PIP_2_: phosphatidylinositol 4,5-bisphosphate
PIP_3_: phosphatidylinositol 3,4,5-trisphosphate
PIPK: phosphatidylinositol phosphate kinase
PIPKIIs: type II phosphatidylinositol phosphate kinases
PIPKIs: type I phosphatidylinositol phosphate kinases
PKC: protein kinase C
PLC: phospholipase C
PR: proline-rich
PTEN: phosphatase and tensin homolog deleted on chromosome 10
Rictor: rapamycin-insensitive companion of mTOR
RTK: receptor tyrosine kinase
SCA: spinocerebellar ataxia
SH2: Src homology 2
SHIP: Src homology 2 containing inositol phosphatase
SKIP: skeletal muscle- and kidney-enriched inositol polyphosphate phosphatase
SYNJ: synaptojanin
TM: transmembrane spanning region
TPIP: transmembrane phosphatase with tensin homology and phosphatase and tensin homolog deleted on chromosome 10 homologous inositol lipid phosphatase
TPTE: transmembrane phosphatase with tensin homology
TRP: transient receptor potential
VSP: voltage-sensing phosphatases
